# Will deeper roots be enough? Engineering drought-resistant crops will entail in-depth understanding of root hydraulic architecture. A Commentary on ‘Root and xylem anatomy varies with root length, root order, soil depth and environment’

**DOI:** 10.1093/aob/mcac081

**Published:** 2022-07-08

**Authors:** Alain Pierret

**Affiliations:** Institute of Ecology and Environmental Sciences of Paris (iEES-Paris- Sorbonne Université, Univ. Paris Est Creteil, IRD, CNRS, INRAE), Department of Agricultural Land Management (DALaM), Vientiane, Lao PDR

**Keywords:** Deep roots, drought resistance, root hydraulic architecture, root conductance, sustainable crop intensification

## Abstract

This article comments on:

Corentin Clément, Hannah M. Schneider, Dorte Bodin Dresbøll, Jonathan P. Lynch and Kristian Thorup-Kristensen, Root and xylem anatomy varies with root length, root order, soil depth and environment in intermediate wheatgrass (Kernza^®^) and alfalfa, Annals of Botany, Volume 130, Issue 3, 1 September 2022, Pages 367–382 https://doi.org/10.1093/aob/mcac058

With the recently emerging awareness of the potential importance of deep root function, there is now a modest but growing research effort devoted to filling the numerous knowledge gaps in our understanding of these out-of-sight, yet functionally essential, plant parts. Such an effort is much needed: while better exploitation of resources from deep soil layers could help drive the sustainable intensification of crop production (Thorup-Kristensen *et al.*, 2020), a number of widely accepted hypotheses and models related to root function rest on a rather frail basis.

As a significant contribution to this effort, the paper by Clément *et al.* (2022) in this issue of the journal explores how different parts of the root system vary in their ability to take up and transport water, revisiting the paradox that despite the presence of roots within deeper soil layers, water-stressed crops often fail to utilize available subsoil water (Passioura, 1991). Potential causes of this paradoxical behaviour that have been investigated so far include: soil structural constraints that induce a sub-optimal exploration of the soil volume; limited transport of soil water to the root due to low unsaturated soil hydraulic conductivities; buildup of osmotic pressure at the soil–root interface; and development of large hydraulic resistance at the root–soil interface as roots shrink and loose contact with the soil when the soil is drying. Indeed, in structured soils (soils whose porosity derives from the arrangement of aggregates of different shapes and sizes), White and Kirkegaard (2010) found that a lack of contact between root and soil induced significant hydraulic resistance at the root–soil interface, explaining why 20 % of subsoil available water remained unused by a mature wheat crop under terminal stress. Based on such observations, White and Kirkegaard (2010) logically suggested that, to overcome drought stress and crop failure, breeding plants that are able to rapidly grow in macropores and branch in softer parts of the soil should be considered. Indeed, it is often assumed that root placement in deep soil layers is the precondition to plants accessing moisture under drought conditions. While such a proposal can hardly be questioned, whether such a root architectural trait would, in itself, suffice to warrant drought tolerance is unclear.

In their paper, Clément *et al*. point out that this paradox could also result from limitations related to root hydraulic properties, as a direct consequence of the anatomy and/or root architecture of roots. Indded, root hydraulic conductance is critical for water transport through the soil–plant–atmosphere continuum and has two separate components: a radial component corresponding to water movement into roots and an axial component that determines water movement along roots. Based on root direct measurements of root axial conductance on root segments and architectural modelling, I and my colleagues previously found significant discrepancies between the hydraulic maturity of primary and lateral roots of maize depending on depth: after 100 d of growth, at soil depths of 0.5 and 1 m, only ~60 and 10 % of main axes respectively had reached their maximum axial hydraulic conductance, compared to ~90 and 80 % of laterals at the same depths (Pierret Doussan and Pagès, 2006; Fig. 1). Such low axial hydraulic conductance may reflect a conservative water use strategy but, depending on how it is distributed within the root system, it also potentially limits the ability of plants to take advantage of subsoil water when they most need it.

**Fig. 1.**
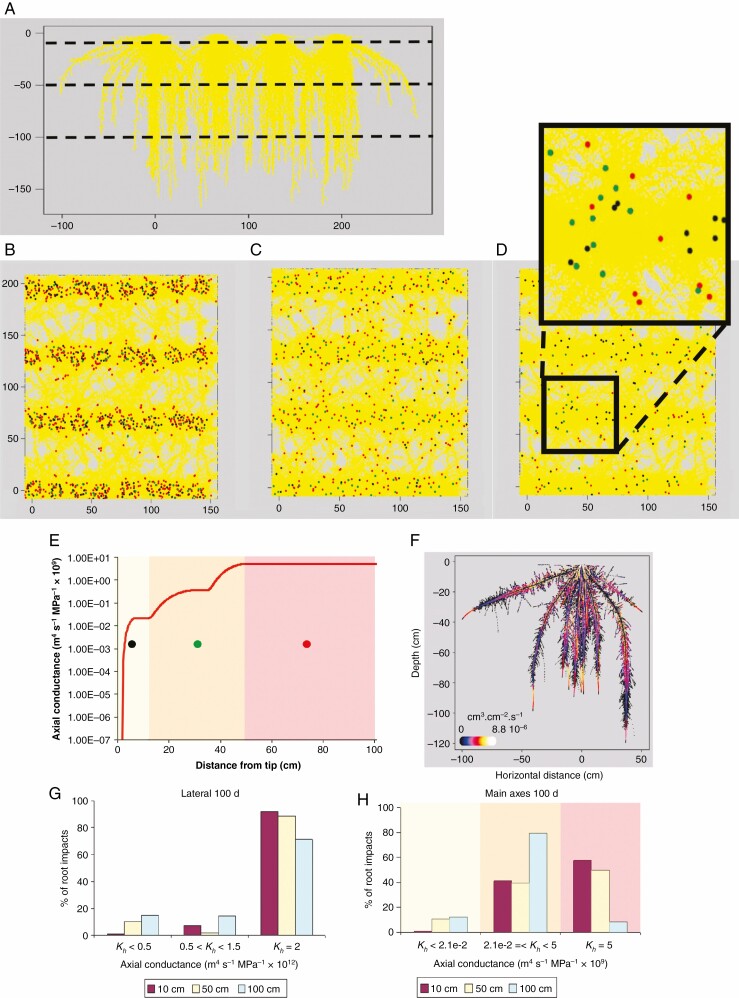
Spatial distribution of root axial conductance (*K*_*h*_) in a population corresponding to a 100-d- old maize crop based on root hydraulic architecture modelling (Doussan *et al.*, 1998). (A) Side view (perpendicular to the direction of sowing rows) of the vertical spread of root systems (horizontal and vertical scales in cm). Horizontal dotted lines indicate depths of the horizontal sections represented in B–D. Root impact densities – positions of root intersections with the considered soil depth – corresponding to the main axes of the simulated crop at soil depths of 10 (B), 50 (C) and 100 (D) cm, respectively (inset shows detail of main axis distribution and colour-coded *K*_*h*_ classes). Yellow outlines represent root systems projected on the horizontal plane. Red, green and black dots reflect the three *K*_*h*_ classes defined in E. Red dots represent hydraulically mature axes, with fully differentiated late metaxylem (LMX) vessels. While a vast majority of laterals were hydraulically mature at all soil depths (G), root populations encompassed increasing amounts of hydraulically immature main axes with increasing soil depth (H). Such hydraulic root architecture induces patchy water uptake rates with a distal–proximal uptake gradient along main axes (F) (redrawn from Doussan *et al*., 1999) and the collecting capacity of the main axes may not match the water influx converging from numerous laterals, particularly at depth, in line with the findings of Clément and co-workers.

To further this anatomical/architectural hypothesis, Clément *et al*. used state-of-the art methodology to assess whether differences in anatomical traits of distal roots could account for differences in their capacity to take up water from deep soil. The authors sampled a set of deep roots from intermediate wheatgrass and alfalfa grown according to three growth modalities, namely hydroponics, soil in the field and in large soil-filled rhizoboxes (allowing root system development down to 4 m). Extracted root segments sectioned using laser ablation were subsequently imaged with a digital camera and analysed using image analysis routines to measure root anatomical features (including number, size and area of individual metaxylem vessels); finally they estimated root axial conductance based on the number and diameter of observed metaxylem vessels.

Despite some degree of variability arising from species and growth conditions, Clément *et al.*’s results clearly highlight a decrease in the number or diameter (or both) of metaxylem vessels along roots in the distal direction, implying that deeper roots have, on average, a lower hydraulic conductance then shallower ones. Overall, these findings also support the idea that water flow capacity is limited by the hydraulic properties of the axial/parent root, as proposed by Pierret *et al.* (2006).

Over the last two decades, the literature record in the field of agronomy has seen the rise of a much-publicized research dealing with the development of so-called soil health indicators (SHIs; Li *et al.*, 2021), typically derived from simple measurements (increasingly relying upon low-cost, widely available technology) of chemical, physical and biological properties of topsoil layers. Amidst this quest for simple answers to complex problems, the paper by Clément and co-workers is a timely piece of evidence and a clear reminder of the actual complexity of the structures and processes that prevail within the Earth’s Critical Zone (Lin, 2010). For example, the topology of root systems follows two broad categories: (1) fibrous (or diffuse, or fasciculate) root systems consisting of a variable number of primary axes, directly connected to the stem, and bearing variable orders of branch roots; and (ii) tap-rooted (or central, or conical) root systems consisting of a central, vertical main root (tap root) along which branching roots originate. Additional sub-types are defined based on the distribution and relative importance of branching root types. Such branching patterns appear to greatly influence root water uptake. Rewald *et al.* (2011) reported evidence that root branching was, over eight successive orders, a determinant of water flux, as a consequence of differences in tissue densities and specific root area (SRA), with first-order roots displaying a significantly higher rate of water uptake than roots of higher branching orders. In turn, these hydraulic differences between root orders have also been found to relate to differences in the root microbiome; using 16S rRNA gene sequencing, King *et al.* (2021) reported that populations of root-associated bacteria differed most from that of bulk soil bacteria in the lowest root orders, in conjunction with higher bacterial abundance and higher representation of taxa associated with carbon mineralization.

While simple concepts such as ‘soil health’, however imperfect, have their merit in that they provide practical benchmarks that can incrementally improve agroecosystems sustainability, the yet-to-be-discovered complexity of roots within the Critical Zone contains the possibility of larger leaps in progress. Delivering such leaps will require targeted physiological and structural research conducted in tandem with breeding programmes. Beyond simple architectural traits such as rooting depth, selection of crops that effectively use subsoil water will probably require, as the study by Clément and co-workers shows, a thorough understanding of the hydraulic architecture and anatomy of root systems, and robust models of root function (Meunier *et al.*, 2019). Finally, more effort should also be put into deciphering root-to-shoot signalling in response to stomatal closure, and the implications of such signalling for root hydraulic architecture selection.
